# Apoptosis and Cell Proliferation Markers in Inflammatory-Fibroproliferative Diseases of the Vessel Wall (Review)

**DOI:** 10.17691/stm2020.12.4.13

**Published:** 2020-08-27

**Authors:** E.A. Klimentova, I.A. Suchkov, A.A. Egorov, R.E. Kalinin

**Affiliations:** Department of Cardiovascular, X-ray Endovascular, Operative Surgery, and Topographic Anatomy; Ryazan State Medical University, 9 Vysokovoltnaya St., Ryazan, 390026, Russia; Professor, Department of Cardiovascular, X-ray Endovascular, Operative Surgery, and Topographic Anatomy; Ryazan State Medical University, 9 Vysokovoltnaya St., Ryazan, 390026, Russia; Doctoral Student, Department of Cardiovascular, X-ray Endovascular, Operative Surgery, and Topographic Anatomy; Ryazan State Medical University, 9 Vysokovoltnaya St., Ryazan, 390026, Russia; Professor, Head of the Department of Cardiovascular, X-ray Endovascular, Operative Surgery, and Topographic Anatomy Ryazan State Medical University, 9 Vysokovoltnaya St., Ryazan, 390026, Russia

**Keywords:** apoptosis, markers of apoptosis, cell proliferation, inflammatory disorders of the vessel wall.

## Abstract

Apoptosis is the main feature of inflammatory-fibroproliferative disorders of the vessel wall. Studies in animal models have shown that smooth muscle cells (SMCs) cultured from endarterectomy specimens from the affected area proliferate more slowly and display higher apoptotic indices than SMCs derived from the normal vessel wall. Apoptotic cells were found in the destabilized atherosclerotic plaques, as well as in the samples with restenosis of the reconstruction area.

Injury to the vessel wall causes two waves of apoptosis. The first wave is the rapid apoptosis in the media that occurs within a few hours after injury and leads to a marked reduction in the number of vascular wall cells. The second wave of apoptosis occurs much later (from several days to weeks) and is limited by the SMCs within the developing neointima. Up to 14% of the neointimal SMCs undergo apoptosis 20 days after balloon angioplasty. Ligation of the external carotid artery in a rabbit model led to a marked decrease in blood flow in the common carotid artery, which correlated with the increased apoptosis of endothelial cells and SMCs.

Angioplasty-induced death of SMCs is regulated by a redox-sensitive signaling pathway, and topical administration of antioxidants can minimize vascular cell loss. On the whole, studies show that apoptosis is prevalent in vascular lesions, controlling the viability of both inflammatory and vascular cells, determining the cellular composition of the vessel wall. The main markers of apoptosis (Fas, Fas ligand, p53, Bcl-2, Bax) and cell proliferation (toll receptor) have been considered in the current review.

## Introduction

Normal vascular cells undergo little apoptosis. It is an important component of the vessel wall remodeling process that occurs during the formation of atherosclerotic and restenotic lesions [1–3]. Apoptotic death is a way to remove infiltrating leukocytes and damaged cells without developing inflammation [4, 5]. It functions properly in the early stages of the disease, and an increase in the apoptotic rate in a later stage of the lesion is associated with insufficient removal of dying cells, the development of secondary necrosis, exacerbation of inflammation in the atherosclerotic plaque (AP).

Initially, the study of the process of apoptosis was carried out in endarterectomy samples from the zone of atherosclerotic and restenotic lesions; it was found in the macrophages, smooth muscle cells (SMCs), T lymphocytes. In the initial stages of atherosclerosis, the activity of cell proliferation is higher than the rate of apoptosis, which causes atherosclerotic plaque formation and development [6, 7]. With the progression of the process, apoptosis prevails in various cells of the AP (SMCs, fibrous cap, etc.), causing its instability and rupture with subsequent thrombosis. Clinical studies of patients with myocardial infarction have demonstrated an increase in apoptotic markers (FADD and caspase-8) in the acute phase of the event [8].

Studies of cell death in vascular lesions were carried out in animal models. In experimental studies, activation of the L-arginine/nitric oxide (NO) metabolite pathway causes regression of atheroma via the induction of macrophage apoptosis. Increased expression of apoptotic genes of Bax proteins, caspase-3, caspase-9, p53, and FAS in unstable plaques was established in rabbit models [9].

Apoptotic cells (SMCs, macrophages, and leukocytes) were found in the samples with restenosis of the reconstruction zone. The analysis of the neointima after percutaneous transluminal balloon angioplasty (PTA) showed that apoptosis reaches its maximum after 2 weeks, while continuous cell proliferation occurs within 12 weeks without an increase in the cell composition. PTA induces a rapid wave of apoptosis of the SMCs in the carotid artery media. Intact vessels and vessels examined immediately after PTA display no signs of apoptosis. However, 0.5–1 h after injury, up to 70% of medial SMCs demonstrate nuclear condensation (an independent indicator of apoptosis), and after 4 h they are no longer detectable [10].

Rapid onset apoptosis has been observed in PTA models with unilateral and bilateral lesions of the rabbit iliac arteries [11]. A big amount of cell loss depended on the intensity of damage in both models, apoptosis being observed more often in the media than in the neointima. Although the quantification of apoptosis in neointimal lesions remains a matter of controversy, studies have demonstrated that the restenotic zone contains a higher SMC density and significantly reduced levels of apoptosis compared to primary atherosclerotic lesions. There is a hypothesis that the activation of the anti-apoptotic gene *Bcl-xL* in neointimal cells can give them relative resistance to the induction of their death due to injury.

The immunohistochemical study of the synthetic shunts after their implantation into the vascular bed showed the presence of apoptotic cells in the intima after 1 month, and in the graft adventitia after 6 months. Until now, the mechanisms and role of macrophages in the distribution of these cells in the vessel walls are not fully known. Macrophages induce Fas upregulation in the cultured SMCs [12–14].

On modeling a venous shunt in experiments on rabbits, Wan et al. [15] showed that the level of apoptosis is highest between the 1^st^ and 3^rd^ days after surgery, while proliferation reaches its maximum on the 7^th^ day. As the restenosis of the venous graft progresses, signs of apoptosis are revealed.

Pro-oncogenic c-Myc controls cell proliferation, apoptosis, tissue remodeling, angiogenesis, cell metabolism, and the production of inflammatory and anti-inflammatory cytokines [16–21]. Steger et al. [22] showed that the use of an antisense oligonucleotide against c-Myc in a mouse venous graft model involves a significant reduction in neointima formation in the postoperative period.

Percutaneous transluminal balloon angioplasty-induced death of SMCs is regulated via redox-sensitive signaling pathways, and local administration of antioxidants can minimize the loss of vascular wall cells [23–29]. Lipophilic statin use causes apoptosis of various types of cells, including SMCs and endothelial cells, while hydrophilic statins (rosuvastatin and pravastatin) do not. Clinical significance of statin-induced apoptosis remains controversial since it can reduce the thickening of the vascular wall in the early stages of atherosclerosis, as well as reduce the formation of neointima in response to damage on the one hand, and on the other hand, it can promote the atherosclerotic plaque destabilization causing acute cardiovascular events [30–34].

## Apoptosis markers

### Fas receptor and its ligand.

One of the main participants in the apoptosis system is the Fas receptor and its ligand. Fas ligand (FasL) is a type II membrane protein that belongs to the tumor necrosis factor family and induces apoptosis through a cognate interaction with the Fas receptor (CD95/Apo-1). The Fas–FasL system is involved in the regulation of physiological cell metabolism. Fas contains a cytoplasmic domain called the death domain (Fas-associated death domain), which is required for transmission of an apoptotic signal. The Fas death domain interacts with the FADD protein. A separate FADD domain binds to procaspase-8. Fas leads to caspase-8 activation through autoproteolysis followed by proteolytic cleavage of other members of the caspase family. Caspase-8 activation and Fas-mediated apoptosis can be blocked by endogenous inhibitory molecules such as FLIP (FLICE-like inhibitory proteins) [35, 36].

Fas-mediated death signaling may involve the activation of anti-apoptotic proteins Bcl-2 and Bcl-XL. Mitochondrial activation results from caspase-8-mediated cleavage of the Bid protein, which is then translocated into the mitochondrial membrane and induces cytochrome release [37].

Fas is expressed on many types of cells, including inflammatory and vascular ones. FasL was found in inflammatory cells and tissues, vascular endothelium, where it played an important role in controlling the extravasation of inflammatory cells. Endogenous FasL mainly functions to suppress inflammatory responses, its non-physiological expression can cause neutrophil infiltration due to the release of IL-1β and neutrophilic chemoattractant after Fas activation. Since SMCs express Fas, and inflammatory cells express FasL, it has been suggested that Fas-mediated apoptosis may promote AP instability. Local delivery of FasL to the carotid arteries of the rats after PTA induced apoptosis in proliferating SMCs [38, 39].

Unlike SMCs, vascular endothelial cells are resistant to Fas-mediated apoptosis under normal conditions. Endothelial cells with Fas on their surface do not undergo apoptosis when exposed to an agonist anti-Fas antibody or when FasL is increased through adenoviral delivery. They remain resistant to Fas-mediated cell death even when activated by interferon-γ, which markedly increases Fas expression. This occurs due to low FasL synthesis on the cell surface, production of endogenous inhibitors of the Fas pathway of death signaling, or FLIP expression, which ensures their survival. Other defense mechanisms may include the involvement of the Bcl-2 family proteins which function as positive and negative regulators of apoptosis. NO production by endothelial cells promotes the nitrosylation and inactivation of the caspases which are required for Fas-mediated cell death. An interesting fact is that NO released from the SMCs of IL-1 stimulated rats promotes Fas expression independently of cGMP [40].

Local administration of TNF-α to arteries inhibits endothelial FasL and induces strong leukocyte infiltration of the vessel wall. TNF-α secretion by activated cells within atheroma can inhibit FasL expression in the adjacent cells of normal endothelium, promoting increased extravasation of leukocytes and wall damage. TNF-α-induced macrophage and T cell infiltration is markedly attenuated when FasL is constitutively expressed in vascular endothelium by adenovirus-mediated gene transfer. Chronic localized immune response is a hallmark of atherogenesis. The observation that FasL is able to inhibit inflammatory responses in the vessel wall suggests that it may have an atheroprotective function [41].

On the other hand, changes in the expression of FasL and Fas may indicate endothelial cell dysfunction, which promotes atherogenesis. For example, oxidized low-density lipoproteins (LDL) can alter the sensitivity of these cells to Fas-mediated apoptosis. Sensitization of Fas-mediated apoptosis correlates with suppression of FLIP and estimates the role of this inhibitor in the natural resistance of endothelial cells to Fas stimulation [41].

English et al. [42] proved that the tissue inhibitor of metalloproteinases-3 (TIMP3) induces apoptosis in human SMCs via caspase-8 and FAS activation. Imanishi et al. [43] showed that a decrease in FLIP synthesis in the media is observed 24 and 48 h after PTA, and its return to the initial level occurs on the 28^th^ day.

Neointimal formation after PTA is the Achilles heel of this type of treatment. PTA results in the immediate loss of SMCs due to apoptosis, followed by their rapid proliferation. It was established [44] that forced expression of FasL through adenoviral delivery *in vivo* reduces neointimal formation after PTA in rabbits and rats.

Luo et al. [45] compared the efficiency of the delivery of FasL and p21 protein genes by their ability to inhibit neointimal hyperplasia in the area of surgery on the carotid arteries of rats. Only Ad-FasL inhibited neointima formation in the immunized rats markedly reducing infiltration by T cells. Local delivery of FasL has two effects on the vessel wall: it induces apoptosis in Fas-bearing SMCs and inhibits the destructive response of T lymphocytes to the cells expressing viral proteins.

On the other hand, the formation of neointimal hyperplasia caused by the simulation of mechanical damage to the femoral artery in normal mice or by FasL or Fas deficiency did not differ, which indicates the existence of other signaling pathways of apoptosis in vessel wall injury [46].

### Toll-like receptors (TLR).

It is a class of cellular receptors with a single transmembrane fragment that recognize microorganisms and activate cellular immune response. Today, 13 types of TLR are known, only 9 types have been found in humans. They are presented on cells of different types — from epithelial to immunocompetent ones. For example, TLR4 is expressed on cardiomyocytes, macrophages, SMCs, and endothelial cells. The known TLR ligands are various bacterial and fungal components including peptidoglycan for TLR2, lipopolysaccharide for TLR4, phagellin for TLR5, etc. Cellular fibronectin produced in response to tissue damage, HSP60 and HSP70 heat shock proteins, LDL, necrotic cells are activators for TLR2 and TLR4. An interesting fact is that human HSP60 requires TLR4 activation to stimulate TNF-α and NO production [47].

The TLR mechanism of action includes signal transduction into the cell nucleus and dimerization, accompanied by altering the conformation of the TIR domain, which binds to the MyD88 adapter molecule required for recruiting IRAK (IL-1 receptor-associated kinase) family kinases [48–51].

Toll-like receptors initiate the activation of nuclear factor (NF-κB), leading to the production of pro-inflammatory cytokines and chemokines such as IL-1α, IL-1β, IL-1Ra, IL-6, IFN-γ, interferon-induced protein-10 (IP-10), inflammatory protein of macrophages (MIP-1β), chemotactic protein-1 of monocytes (MCP-1), IL-8. These inflammatory mediators can exert various atherogenic effects including the expression of adhesion molecules on endothelial cells, SMC proliferation, immune cell activation, and the stimulation of the acute phase response. There is evidence of the involvement of NF-κB in the development of atherosclerosis [52–56]. Michelsen et al. [57] reported that macrophage-specific inhibition of the NF-κB pathway leads to a more severe course of atherosclerosis in mice (possibly due to a decrease in IL-10 production by macrophages).

In recent years, evidence has emerged regarding TLR involvement in the pathogenesis of atherosclerosis. Normal arteries displayed very low levels of all TLRs with the exception of a relatively higher level of TLR4 mRNA. In the atherosclerotic lesion area, TLR1, TLR2, and TLR4 were increased threefold, their synthesis is carried out predominantly by endothelial cells and macrophages. In a mouse model with a TLR4/ApoE deficiency, a lower formation of atherosclerotic plaques with a stable phenotype was displayed due to the reduced levels of IL-12, MCP-1, etc. Disabling MyD88, an adapter molecule of the TLR signaling pathway in ApoE–/– mice, decreased the number of atherosclerotic lesions of the aorta by 40–65% and significantly reduced the formation of cyclooxygenase-2 in the atherosclerotic plaques. Thus, TLR4 and MyD88 are key players in the progression of atherosclerosis [58].

Toll-like receptor-2 promotes SMC migration by increasing IL-6 production. Numerous studies [59–62] show that the receptor agonists stimulate the migration of SMCs, but not cell proliferation and viability. Deficiency of the receptor or its inhibition by an antibody inhibits TLR2 agonist-induced SMC migration and IL-6 production mediated by p38 mitogen-associated protein kinase and extracellular kinase 1 and 2 signals. These signaling pathways work together to activate cAMP and subsequent IL-6 production, which, in turn, contributes to SMC migration. The use of antibodies against IL-6 disrupted TLR2-mediated SMC migration. Schoneveld et al. [63] in their experiment demonstrate the local application of a specific ligand to stimulate TLR2 on the femoral artery of mice, inducing the formation of vascular neointimal hyperplasia. Kobayashi et al. [64] found in animal models that infection with *Porphyromonas gingivalis* can promote neointima formation after arterial injury by signaling through TLR2. Zhang et al. showed [65] that palmitate-induced generation of reactive oxygen species and SMC apoptosis, accompanied by the inhibition of the caspase-3, caspase-9, and p53 expression, as well as Bcl-2 expression restoration, were significantly blocked in mice lacking TLR4.

In the patients with *Asp299Gly* TLR4 gene polymorphism, less intima/media thickness in the carotid artery was observed than in healthy volunteers. The patients with this polymorphism suffering from ischemic heart disease demonstrated greater benefit from treatment with pravastatin (HMG-CoA reductase inhibitor) compared to healthy volunteers [66].

### Proteins of the Bcl-2 family.

In many situations, cell viability is controlled by modulation of the levels of the Bcl-2 family proteins. Some of these proteins (Bcl-2, Bcl-XL, and A1) act as inhibitors of apoptosis, while others (Bax, Bad, and Bid) are modulators. The ratio of these proteins is believed to control cell survival [67–70].

As a rule, proteins of the Bcl-2 family function at the mitochondrial level, control cell viability, as well as the movement and action of cytochrome C, which accelerates apoptotic cell death. When the Apaf-1 protein is released from mitochondria, cytochrome C binds to it and pro-caspase-9, which results in the proteolytic activation of this caspase. Then caspase-9 proteolytically activates downstream caspases and cell death occurs. The Bcl-XL protein forms an ion channel in the synthetic lipid membranes, regulating mitochondrial function and membrane permeability [71–74].

The Bax protein level is elevated in the SMCs of human atherosclerotic plaques where apoptosis occurs at a high rate [75]. On the contrary, the protective factor for Bcl-XL apoptosis is detected in normal SMCs of rat media, and its expression is rapidly decreased after PTA. A decrease in Bcl-XL occurs 1 h after injury in the media. Increased Bcl-XL levels were observed in the atheromatous lesions of the human intima in comparison to the media cells. When an antisense oligonucleotide directed against Bcl-XL was injected into the balloon-damaged carotid arteries of a rabbit 4 weeks after the procedure, Bcl-XL expression was suppressed and intimal cell apoptosis occurred at an increased rate. This resulted in a 56% reduction in intimal thickness. The studies have demonstrated that the Bcl-2 family proteins are functionally significant in the vessel wall and differential expression of Bcl-XL can contribute to the differential sensitivity of medial and neointimal cells to rapid apoptosis [76–78].

### p53 transcription factor.

The fact that the p53 transcription factor activates Bax expression and inhibits the Bcl-2 protein, which leads to conditions promoting apoptosis, may be significant in the disease of the vessel wall [79, 80]. Activation of p53 also increases Fas expression on the cell surface of human SMCs, stimulating transport from the Golgi apparatus. The antiproliferative activity of p53 is mediated by its ability to induce a p21 cyclin-dependent kinase inhibitor. The p53 factor can induce SMC apoptosis *in vitro* and inhibit SMC proliferation *in vitro* and *in vivo*. It is accumulated in atherosclerotic lesions, and its ability to initiate apoptosis in these lesions may depend on interaction with MDM2 protein, a regulator of p53 stability [81–84]. It was reported [85] that the cytomegalovirus gene counteracts p53-induced apoptosis of SMCs. Mice deficient in this protein, developed accelerated atherosclerotic aortic lesions when fed a high-fat diet compared to healthy mice. However, the increased lesion size due to p53 deficiency appears to be the result of an increase in the rate of cell proliferation rather than a decrease in apoptosis. Thus, p53-independent apoptotic mechanisms may predominate in the atherogenic vessel wall [86, 87].

The functions of the Bcl-2 family proteins also include the regulation of the viability of endothelial cells. For example, endothelial cell survival is facilitated by vascular endothelial growth factor (VEGF), which is associated with increased Bcl-2 levels. Protein kinase activation is required for VEGF-mediated endothelial cell survival and VEGF-mediated induction of Bcl-2. TNF-α-induced endothelial cell death includes Bcl-2 degradation [88, 89].

Nitric oxide, synthesized from L-arginine with the help of NO synthases, is a small, lipophilic, diffusing, highly reactive molecule with a dichotomous regulatory role in many biological events under physiological and pathological conditions [90]. NO can promote apoptosis in some cells and inhibit apoptosis in others. The local cellular environment plays a dynamic role in determining the nature of these types of regulation [91, 92]. Important components of the microenvironment are as follows: the redox state of cells, glutathione, the presence of other oxygen- and nitrogen-centered radicals. High NO production acts as a pro-apoptotic modulator, activating proteases of the caspase family through the mitochondrial cytochrome C release into the cytosol, p53 protein up-regulation, activation of the JNK/SAPK signaling pathway, by altering the expression of the apoptosis-associated proteins of the Bcl-2 family. Nevertheless, low or physiological concentrations of NO inhibit cell apoptosis induced by Fas, LDL, and cytokines. The antiapoptotic mechanism is associated with gene transcription of protective proteins (Bcl-2, heat shock protein, cyclooxygenase-2) and direct inhibition of effector caspases by S-nitrosylation of the thiol group of cysteine in their catalytic site [93–97]. Recent studies on hypercholesterolemic rabbits have demonstrated that activation of the L-arginine/NO pathway causes atheroma regression, most likely through the induction of macrophage apoptosis. Dubey et al. [98] demonstrated the decisive role of inducible NO synthase in neutrophil apoptosis through enhanced generation of reactive oxygen species and caspase-8-mediated activation of the mitochondrial death pathway. NO increases Fas expression on SMCs, which enhances their apoptosis under these conditions [99, 100].

## Conclusion

Cell apoptosis is widespread in inflammatory-fibroproliferative lesions of the vessel wall. Cell death can affect lesion volume, plaque stability, inflammation, and endothelial integrity. Recent research has identified numerous regulatory pathways that could potentially control vascular cell viability. Understanding these regulatory networks at the molecular level will help to form the understanding of the pathogenesis of vascular diseases and lead to the development of new treatments for these disorders.
